# AI-Enabled Patient Profiling for Personalized Nursing Care and Health Support: Scoping Review

**DOI:** 10.2196/103362

**Published:** 2026-10-29

**Authors:** Jingyan Zhang, Fengyi Zhang, Youpeng Guo, Jingwen Zhang, Hongmei Zhong, Xuemei Guo, Min Tan

**Affiliations:** 1 Affiliated Hospital of North Sichuan Medical College Nanchong, Sichuan China; 2 Xiangjiang Laboratory Changsha, Hunan China; 3 Key Laboratory of Digital-Intelligent Disease Surveillance and Health Governance North Sichuan Medical College Nanchong China

**Keywords:** artificial intelligence, AI, machine learning, nursing care, patient profiling, personalized care, digital health, clinical decision support, patient-centered care

## Abstract

**Background:**

AI-enabled patient profiling has been proposed to organize heterogeneous patient data and support personalized nursing care and health support. Given the growing use of AI, machine learning, large language models (LLMs), digital phenotyping, and digital twins in health care, it is important to clarify how patient profiling has been conceptualized, applied, and evaluated in nursing-relevant contexts.

**Objective:**

This scoping review aimed to examine and report the available evidence on AI-enabled patient profiling for personalized nursing care and health support.

**Methods:**

This scoping review was conducted using the Joanna Briggs Institute methodological framework and the PRISMA-ScR (Preferred Reporting Items for Systematic Reviews and Meta-Analyses Extension for Scoping Reviews). Chinese and English databases were searched from inception to May 18, 2026, including China National Knowledge Infrastructure, Wanfang Data, VIP Database, Chinese Biomedical Literature Database, PubMed, CINAHL, Web of Science, and Embase. Eligible studies used AI or computational intelligence methods to construct, classify, apply, validate, or evaluate patient profiles or related patient representations relevant to personalized nursing care or health support. Two reviewers independently screened records, assessed full texts, and charted data using a predefined form. Data were synthesized descriptively and narratively using an input-process-output framework.

**Results:**

A total of 61 studies were included. AI-enabled patient profiling was represented through patient profiles, patient or user personas, digital phenotypes, patient segments, clinical subtypes, digital twins, and LLM-enabled personalization. Included studies used diverse data sources, including electronic health records, nursing records, patient-reported outcomes, questionnaires, mobile app data, wearable and sensor data, engagement logs, and textual or conversational data. Common AI approaches included machine learning, clustering, deep learning, natural language processing, recommender systems, knowledge graphs, digital twin modeling, and LLMs. Applications focused on personalized education, self-management support, rehabilitation, chronic disease management, remote monitoring, decision support, follow-up care, and shared decision-making. Most studies evaluated algorithmic performance, feasibility, usability, or system development, whereas fewer studies examined profile validity, actionability, equity, workflow integration, or long-term nursing-sensitive and patient-centered outcomes.

**Conclusions:**

Existing evidence positions AI-enabled patient profiling as an emerging data-to-action mechanism for personalized nursing care and health support rather than as a mature clinical intervention. Future research should strengthen conceptual clarity, validate the profile, establish explicit pathways linking profiles to actions, incorporate nurse and patient involvement, assess equity and safety, and evaluate implementation in real-world nursing settings.

**Trial Registration:**

OSF Registry mey4u; https://osf.io/mey4u

## Introduction

### Background

AI, machine learning, and large language models (LLMs) are increasingly used in health care to analyze clinical, behavioral, and patient-generated data; support risk stratification; inform decision-making; and adapt communication for clinical or patient-facing contexts [1,2]. In nursing, AI has been identified as a priority for research, education, leadership, and practice, but current evidence remains concentrated on early technology development, model testing, and feasibility work rather than sustained implementation or measurable effects on practice [3,4].

This development intersects with a broader shift toward personalized and person-centered care. Precision medicine emphasizes tailoring prevention and treatment by integrating biological, clinical, and contextual data [5], whereas person-centered care emphasizes patients’ values, preferences, capabilities, and lived experience [6]. For nursing, the practical question is therefore not only whether data-driven tools can classify patients, but also whether they can support holistic assessment, care planning, education, follow-up, and evaluation without reducing patients to isolated risks or diagnostic labels [7].

Patient profiling offers 1 possible mechanism for connecting heterogeneous patient information with individualized care. Related concepts include patient profiles, user profiles, patient personas, patient segmentation, and care needs profiles. Personas can represent patient perspectives in user-centered service or intervention design [8], whereas patient segmentation can support integrated care by targeting support to groups with shared needs or characteristics [9].

Digital and computational approaches have broadened profiling beyond static descriptors. Digital phenotyping uses data from smartphones, wearables, and other technologies to characterize behavior, functioning, and health states [10], and machine learning is increasingly used to identify these patterns [11]. Digital twins and LLM-enabled systems extend profiling into simulation, decision support, and personalized communication, while also raising questions about validity, interpretability, privacy, and accountability [2,12].

Despite these developments, the evidence remains fragmented across nursing, informatics, rehabilitation, chronic disease management, behavioral health, and digital health research. Existing nursing reviews have mapped broad AI applications, including prediction, monitoring, decision support, documentation, and communication technologies [3,4], whereas other reviews have examined digital phenotyping or personas separately [8,11]. Less is known about how AI-enabled profiling connects patient-related data with personalized nursing assessment, education, self-management, rehabilitation, follow-up, and health support actions.

Given the emerging and heterogeneous nature of this field, a scoping review was appropriate to clarify key concepts and identify knowledge gaps.

### Review Questions

This review addressed the following questions: (1) How is AI-enabled patient profiling conceptualized? (2) Which data sources and AI methods are used? (3) How are the profiles applied in nursing and health support? and (4) How are the profiles evaluated?

## Methods

### Overview

This scoping review mapped evidence on AI-enabled patient profiling for personalized nursing and health support. A scoping review design was appropriate because the topic is broad; conceptually heterogeneous; and includes diverse study designs, technologies, and application contexts [13-15]. The review followed the Joanna Briggs Institute methodology and the PRISMA-ScR (Preferred Reporting Items for Systematic Reviews and Meta-Analyses Extension for Scoping Reviews) reporting guidance [16,17].

### Protocol and Registration

The review protocol was retrospectively registered on the OSF after study selection and data charting had commenced [18]; therefore, registration should be interpreted as retrospective documentation rather than prospective registration. After retrospective registration, no changes were made to the final database sources, search date, core eligibility criteria, or main data-charting domains. Minor refinements were limited to clarifying operational coding labels during synthesis, including nursing relevance levels and evidence maturity ratings, and these refinements did not alter the included study set. Full search strategies; the data-charting form; full-text exclusion reasons, study characteristics, and analytic coding; and the PRISMA-ScR checklist are provided in Multimedia Appendix 1, Multimedia Appendix 2, Multimedia Appendix 3 [19-79], and Multimedia Appendix 4, respectively.

### Information Sources

Chinese and English databases were searched from inception to May 18, 2026. Chinese databases included the China National Knowledge Infrastructure (CNKI), Wanfang Data, VIP Database, and Chinese Biomedical Literature Database (CBM). English databases included PubMed, CINAHL, Web of Science, and Embase. No publication date restriction was applied. Limits were not used for study design or publication year. Records were eligible only when published in Chinese or English and when the publication type met the source criteria; reviews, protocols, editorials, commentaries, conference abstracts, preprints, and records with insufficient information for data charting were excluded during screening or full-text assessment. Preliminary scoping searches were conducted in PubMed, CINAHL, and Web of Science to identify terminology used in titles, abstracts, and indexing records. Final search terms were refined through discussion by the review team. An information specialist or librarian was not formally consulted.

Backward and forward citation searching was not performed; study identification relied exclusively on the predefined electronic database searches.

### Search Strategy

The search strategy combined 3 concept blocks using Boolean operators: AI terms, patient profiling terms, and nursing or personalized care terms. Within each block, synonyms were combined with OR; the 3 blocks were then combined with AND. The AI block included terms such as “generative AI,” “large language model,” ChatGPT, “artificial intelligence,” “machine learning,” and “deep learning.” The profiling block included terms such as persona, “patient persona,” “user persona,” “patient profiling,” “patient profile,” “patient segmentation,” “user modeling,” and “patient typology.” The nursing or personalized care block included terms such as nurs*, “personalized care,” “individualized care,” “precision nursing,” “precision care,” and “tailored intervention.” Quotation marks were used for exact phrases where supported by the database, and truncation was used for nurs* to capture nursing-related variants. Controlled vocabulary terms were used only where they directly corresponded to the original search concepts, including AI, machine learning, deep learning, LLMs, generative AI, and nursing care in PubMed. Terms were adapted for each database according to database-specific syntax and searchable fields. The exact database-specific search strings are provided in Multimedia Appendix 1.

### Eligibility Criteria

#### Population and Context

Eligibility criteria were structured using the population, concept, and context framework recommended for scoping reviews [16]. The population comprised patients, health service users, caregivers, or individuals receiving health-related support. The context comprised nursing or health support, including patient education, self-management, rehabilitation, chronic disease management, remote monitoring, follow-up care, decision support, shared decision-making, digital health, mobile health, and telehealth. Studies focused only on professionals or students were excluded unless they addressed patient profiling for patient care or personalized health support.

#### Concept

The concept of interest was AI-enabled patient profiling, defined as the use of patient-related data and AI or computational intelligence methods to construct, classify, represent, apply, or evaluate individualized or group-based patient representations for personalized nursing or health support. Relevant concepts included patient profiles, personas, digital phenotypes, segmentation, clinical subtypes, risk or care needs profiles, digital twins, individualized patient models, and personalized recommendations.

### Types of Sources

Studies were included if they (1) used AI, machine learning, deep learning, natural language processing, LLMs, recommender systems, knowledge graphs, clustering, digital twin modeling, or related computational intelligence methods; (2) constructed, classified, applied, validated, or evaluated patient profiles or related representations; and (3) were relevant to personalized nursing, patient education, self-management, rehabilitation, follow-up, decision support, or other health support applications.

Studies were excluded if they addressed diagnosis or outcome prediction without patient profiling or related representations; did not involve AI or computational intelligence; lacked relevance to nursing or health support; focused only on virtual patients for professional education; were reviews, protocols, editorials, commentaries, conference abstracts, or preprints; or provided insufficient information for data charting.

### Conceptual Boundary

A conceptual boundary was applied to distinguish AI-enabled patient profiling from adjacent forms of prediction, personalization, and persona development (Table 1).

**Table 1:** Conceptual boundary of AI-enabled patient profiling.

Evidence types	Inclusion decision	Rationale
AI-generated patient representation linked to nursing or health support action	Included	This was core AI-enabled patient profiling evidence because patient-related data are translated into an interpretable profile, segment, phenotype, model, or recommendation for care support.
Prediction model with an interpretable profile, subgroup, or care implication	Included when linked to profiling	This was included only when the output supports patient representation, subgroup identification, or tailored support rather than prediction alone.
Diagnosis or outcome prediction without patient profile representation	Excluded	Prediction alone was not considered patient profiling when it did not produce a profile, segment, phenotype, or action-oriented representation.
Large language model–generated education or communication without patient data–based profiling	Excluded or treated as borderline	Personalization alone was insufficient unless patient information was used to construct or apply a profile-related representation.
Persona studies without AI or computational methods	Excluded	Personas were relevant only when they were developed, validated, or applied using AI or computational intelligence methods.

### Selection of Sources of Evidence

All records were imported into EndNote (Clarivate) for automatic deduplication and manual checking. Chinese- and English-language records were managed separately, and potential duplicate records were checked using title, author, year, journal, and DOI information where available. Two reviewers independently screened titles and abstracts, retrieved potentially relevant full texts, and independently assessed final eligibility. Disagreements were resolved through discussion and consensus, with consultation with another team member when needed. Full-text exclusion reasons are provided in Multimedia Appendix 3. Selection was reported using the PRISMA-ScR flow structure [17].

### Data-Charting Process

A standardized data-charting form was developed from the review aim, research questions, and eligibility criteria. The form was piloted on included studies and refined before formal extraction [16]. Data charting was performed independently and cross-checked by another reviewer for accuracy and completeness. Discrepancies were resolved through consensus. The data-charting form and detailed study characteristics are provided in Multimedia Appendices 2 and 3.

### Data Items

Extracted items included bibliographic details, design, population or setting, sample or data size, context, profiling concept, data sources, AI methods, profiling outputs, nursing or health support applications, evaluation approaches, nursing relevance, and ethical or implementation issues.

Nursing relevance levels were assigned during data charting and cross-checked during synthesis. Disagreements were resolved through discussion, with attention to whether profiling outputs were linked to nursing assessment, education, monitoring, follow-up, rehabilitation, care planning, or decision support.

### Critical Appraisal of Individual Sources of Evidence

Formal critical appraisal was not conducted because the review mapped concepts, methods, applications, and gaps rather than intervention effectiveness [15].

### Synthesis of Results

Data were synthesized using descriptive numerical summaries and narrative synthesis, as recommended for scoping reviews of heterogeneous evidence [14]. Frequencies summarized publication characteristics, evidence relevance levels, profiling concepts, AI methods, and application contexts. Because categories were not mutually exclusive, individual studies could contribute to more than 1 category.

Narrative synthesis was guided by an input-process-output framework. Inputs referred to patient-related data, including electronic health records, patient-reported outcomes, questionnaires, behavioral data, mobile app data, wearable and sensor data, and text or conversational data. Processes referred to AI or computational methods, including machine learning, clustering, deep learning, natural language processing, recommender systems, knowledge graphs, digital twin modeling, and LLMs. Outputs referred to profiles, personas, digital phenotypes, segments, clinical subtypes, care needs or risk profiles, individualized models, digital twins, and personalized recommendations. The synthesis focused on conceptual forms, data sources and methods, applications, and validation and implementation gaps.

To construct the evidence gap map, included studies were cross-tabulated by profiling form and health support context. For visualization only, nursing relevance was mapped as level 3 for 0, level 2 for 0.5, and level 1 for 1. The mapped values were unitless coding scores and did not represent measurement units; when multiple studies occupied the same cell, the mean mapped value determined the bubble color. This color scale was not used as a methodological quality or certainty-of-evidence measure.

### Ethical Considerations

Ethics approval was not required because this review synthesized data from previously published studies and did not involve direct contact with human participants or the collection of primary individual-level data.

## Results

### Included Studies

Database searching identified 1172 records: PubMed (n=131, 11.2%), CINAHL (n=40, 3.4%), Web of Science (n=707, 60.3%), Embase (n=265, 22.6%), CNKI (n=10, 0.9%), Wanfang Data (n=10, 0.9%), VIP Database (n=5, 0.4%), and CBM (n=4, 0.3%). After 106 duplicate records were removed, 1066 records remained for title and abstract screening. During title and abstract screening, 960 records were excluded as not relevant to AI, patient profiling, personalized nursing, or health support. The remaining 106 reports were retrieved and assessed for full-text eligibility. After full-text assessment, 45 reports were excluded for specified reasons, and 61 studies were included in the review. The study selection process is presented in Figure 1.

**Figure 1:**
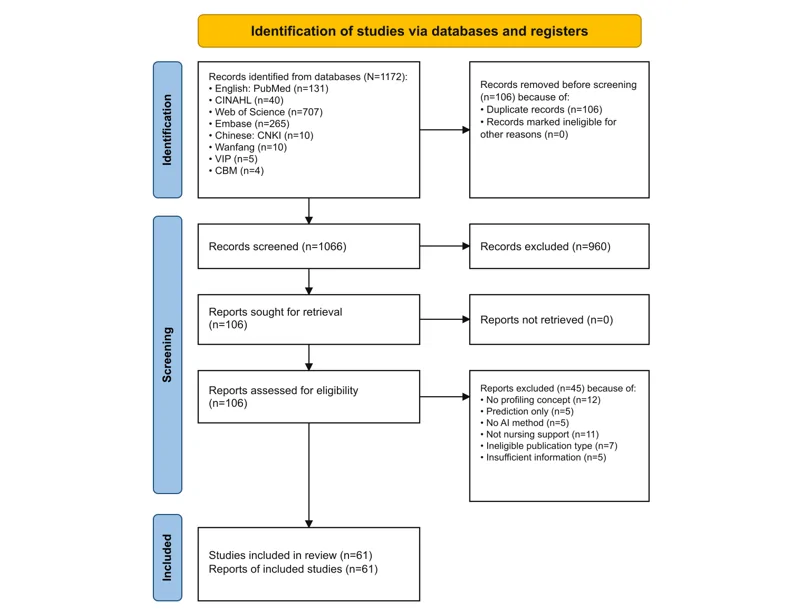
PRISMA-ScR (Preferred Reporting Items for Systematic Reviews and Meta-Analyses Extension for Scoping Reviews) flow diagram of study selection. CBM: Chinese Biomedical Literature Database; CNKI: China National Knowledge Infrastructure.

### Characteristics of Included Studies

The included studies addressed chronic disease management, rehabilitation, cancer care, cardiovascular care, diabetes management, mental health support, older adult care, perioperative and reconstructive care, remote monitoring, digital health, health education, and self-management. For example, 1 study applied an intelligent continuous care model during skin expansion in auricular reconstruction [80]. Designs included randomized and quasiexperimental studies, pre-post studies, feasibility and usability studies, observational and retrospective studies, system development studies, mixed methods studies, and methodological studies. Some studies explicitly addressed nursing functions such as assessment, symptom monitoring, education, adherence support, and follow-up [19]. Others addressed care planning, decision support, engagement, or individualized intervention delivery that was transferable to nursing and health support [20,21]. Detailed characteristics are provided in Multimedia Appendix 3.

Additional included studies represented eating disorder assessment, pre-exposure prophylaxis (PrEP) adherence support, type 1 diabetes phenotyping, precision symptom management, assisted living, sleep apnea follow-up, remote health monitoring, dietary lapse prediction, digital twins, emergency nursing heart failure subtyping, home-based care for older adults, low back pain stratification, behavioral change recommender systems, poststroke smart home monitoring, health literacy profiling, intelligent whole-process health education for day surgery, eHealth user profiling, cognitive health app design, AI-generated self-management guidance, digital mental health recommendation, smoking cessation messaging, medication management, fibromyalgia clustering, oncology care with LLMs, activity-based behavior modeling, embedded user profile service recommendation, and geriatric care plan revision [51-79].

To clarify the degree to which the evidence related to nursing practice, the included studies were coded into 3 levels of nursing relevance before narrative synthesis. Level 1 indicated direct nursing or nurse-relevant care support evidence; level 2 indicated transferable health support evidence; and level 3 indicated methodological profiling evidence with plausible relevance to future personalized health support. These levels were used to interpret applicability rather than to assess methodological quality. The distribution and operational definitions of these levels are summarized in Table 2.

**Table 2:** Nursing relevance levels of included evidence.

Levels	Operational definitions	Number of studies	Representative examples
Level 1	Direct nursing or nurse-relevant care support evidence addressing nursing practice, nursing-sensitive outcomes, nurse-led interventions, nursing decision support, or direct care processes	31	[19,20,80]
Level 2	Transferable health support evidence addressing education, rehabilitation, self-management, follow-up, remote monitoring, or shared decision-making without explicit nursing leadership	15	[21-24]
Level 3	Methodological profiling evidence focused primarily on AI modeling, segmentation, phenotyping, or representation learning, with plausible relevance to personalized nursing or health support	15	[25-27,81]

On the basis of the study-level coding in Multimedia Appendix 3, 31 studies were classified as level 1, 15 as level 2, and 15 as level 3. Because not all level 1 studies were nurse-led, this category was interpreted as directly relevant to nursing or care support processes rather than as exclusively nursing-led evidence. Study-level mapping of profiling concepts, AI methods, nursing or health support applications, evaluation approaches, and evidence relevance levels is provided in Multimedia Appendix 3. The most common contexts were clinical nursing or care support (n=17), digital health or personalized health support (n=12), older adult or long-term care (n=10), and chronic disease or self-management (n=10). The most frequent profiling approaches were patient profile, user model, or personalized patient model approaches (n=42), followed by segmentation or clinical subtyping (n=11), LLM-enabled personalization (n=4), digital phenotyping (n=3), digital twin or individualized models (n=3), and personas (n=3). Common AI approaches were machine learning or clustering (n=26), computational modeling and personalization (n=20), recommender or decision support systems (n=8), LLMs or generative AI (n=6), digital twin modeling (n=3), sensor or Internet of Things analytics (n=3), natural language processing or text mining (n=2), and knowledge graphs (n=1).

### Evidence Gap Map

Figure 2 maps the distribution of included studies across profiling forms and health support contexts. Bubble size represents the number of studies in each profiling form-context combination, and bubble color reflects nursing relevance. Evidence was concentrated in patient profile or model approaches, particularly across clinical care, digital health, chronic disease, and older adult care contexts. In contrast, patient or user persona, digital twin or individual model, and LLM-enabled personalization approaches were represented in fewer contexts. Blank cells indicate profiling form-context combinations for which no included studies were identified.

**Figure 2:**
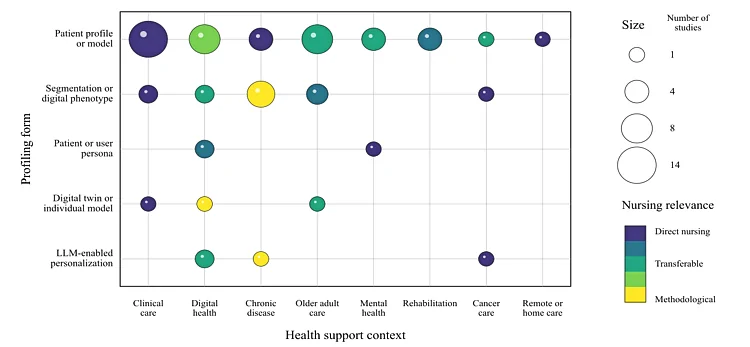
Evidence gap map of AI-enabled patient profiling across profiling forms and health support contexts. Bubble size represents the number of included studies. Bubble color represents nursing relevance; when multiple studies occupied the same cell, the mean nursing relevance level was used for color encoding. Blank cells indicate that no included study was identified for that combination. LLM: large language model.

### Review Findings

#### Conceptual Forms of AI-Enabled Patient Profiling

Included studies used diverse and overlapping terms for AI-enabled patient profiling, with differences in granularity, stability, and actionability. Examples included the use of nursing knowledge and machine learning to identify high-need primary care patients [21], data-driven persona validation [27], digital engagement phenotypes in diabetes prevention [26], and digital twins for shared decision-making in knee osteoarthritis care [22]. Across these terms, the shared function was to transform patient-related information into structured representations that could support individualized assessment, education, decision-making, or health support. These conceptual forms and their levels of representation are summarized in Table 3.

**Table 3:** Conceptual forms of AI-enabled patient profiling.

Profiling forms	Core meaning	Levels of representation
Patient profile or profiling	A structured representation of individual patient characteristics used to support personalized care or health support	Individual-level
Patient or user persona	A typical user or patient representation that reflects needs, preferences, barriers, or engagement patterns	Group-level or archetype
Digital phenotype	A behavioral, physiological, or interaction pattern captured through digital technologies	Dynamic individual- or group-level
Patient segmentation or clinical subtype	A data-driven subgroup with shared risks, needs, behaviors, or response patterns	Group-level
Digital twin	A dynamic individualized model used for simulation, prediction, or shared decision-making	Dynamic individual-level
LLM^a^-enabled personalization	The use of LLMs to interpret patient information or generate tailored communication or support	Individualized communication or recommendation

^a^LLM: large language model.

Patient profiles were structured representations based on demographic, clinical, behavioral, psychosocial, or contextual information [19,80]. Personas translated patient needs, preferences, barriers, and engagement patterns into user-centered representations for intervention design [29,30]. Digital phenotypes captured behavioral, physiological, or interaction patterns generated through digital technologies [31,32]. Segmentation and clinical subtyping classified patients into groups with distinct risks, phenotypes, or care needs [33,34,81]. Digital twins extended profiling toward dynamic simulation and shared decision-making [22], whereas LLM-enabled personalization emerged as a means of interpreting unstructured information and generating tailored communication [35-37].

#### Data Sources and AI Methods Used to Construct Patient Profiles

AI-enabled patient profiles used heterogeneous and often multimodal data. Clinical data, including electronic health records, diagnoses, medications, laboratory results, nursing records, and service use data, supported risk profiles, clinical subtypes, and patient segments [19,20,34]. Patient-reported outcomes and questionnaires captured symptoms, quality of life, self-efficacy, health literacy, preferences, and perceived needs [25,30,38]. Behavioral, engagement, wearable, sensor, textual, and conversational data enabled dynamic profiling for self-management, remote monitoring, rehabilitation, older adult care, and communication support [37,39,40].

Methods reflected the intended profiling purpose. Unsupervised learning and clustering were used to identify subgroups or phenotypes [31]. Supervised machine learning classified risk, adherence, or recommendation categories, while deep learning and natural language processing supported sequential, multimodal, or text-based profiling [22,28,41]. Recommender systems, knowledge graphs, decision support systems, digital twins, and LLMs linked patient features to tailored education, nursing recommendations, caregiver support, shared decision-making, or conversational support [37,42,43]. Figure 3 summarizes the input-process-output framework linking patient-related data, AI processing, profile outputs, nurse mediation, and personalized support actions, with cross-cutting evaluation and governance.

**Figure 3:**
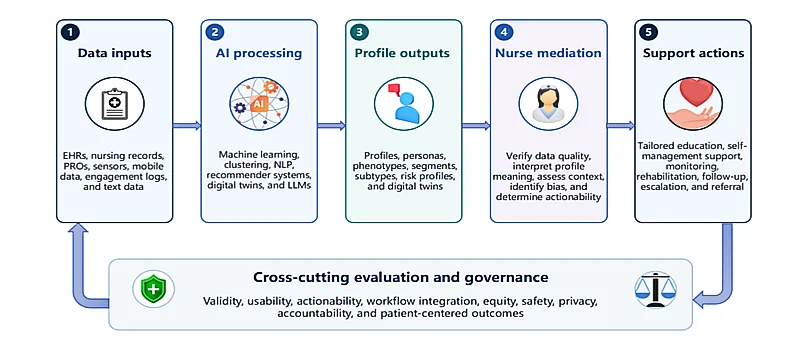
Input-process-output framework for AI-enabled patient profiling in personalized nursing and health support. Patient-related data are processed using AI methods to generate profile outputs, which require nurse mediation before being translated into personalized support actions. Cross-cutting evaluation and governance should address validity, usability, actionability, workflow integration, equity, safety, privacy, accountability, and patient-centered outcomes. EHR: electronic health record; LLM: large language model; NLP: natural language processing; PRO: patient-reported outcome.

### Applications in Personalized Nursing and Health Support

AI-enabled patient profiling was applied to personalized education, self-management, rehabilitation, chronic disease management, remote monitoring, decision support, follow-up, and shared decision-making. In education, profiles informed the content, format, timing, or language of support according to patient characteristics, health literacy, preferences, or communication needs. Examples included tailored smoking cessation messaging [42], machine learning–based personalization of web-based health content [44], a voice-based diet assistant for caregivers of people with Alzheimer disease and related dementias [43], personas for eHealth self-management among people living with HIV [30], and emerging LLM or multilingual care agent approaches to personalized information support [37,45].

Profiling also supported self-management and behavioral tailoring. Decision support and recommender systems personalized self-management support for congestive heart failure, web-based content, smoking cessation, and obesity interventions [24,32,42,44]. Dynamic profiling and personalized messaging supported adherence to cardiac rehabilitation [25], while patient profiling and adherence prediction informed personalized telephone follow-up in obstructive sleep apnea care [23]. Rehabilitation examples included a patient profiling–based rehabilitation nursing platform for spinal cord injury [46], unsupervised profiling of recovery needs after a mild stroke [38], and digital twin decision support for shared decision-making in knee osteoarthritis care [22].

In chronic disease management and remote monitoring, profiles derived from clinical, behavioral, or sensor data supported risk identification, engagement tracking, and recognition of support needs. Digital engagement phenotypes informed tailoring of diabetes prevention and obesity interventions [26,32]. Ecologic momentary assessment supported personalized timing of physical activity [47], and in-home conversational or behavioral monitoring informed follow-up for daily functioning [40]. Decision support and segmentation studies identified high-need patients or groups that could be mapped to care plans, nursing recommendations, postacute services, or home health support [34,48].

Overall, the applications followed a profile-to-action logic in which patient data were translated into patient representations and then into tailored education, monitoring, rehabilitation, decision support, follow-up, or shared decision-making. Table 4 summarizes the main profile-to-action pathways and the maturity of the available evidence.

**Table 4:** Evidence maturity of profile-to-action pathwaysa.

Profile-to-action pathways	Technical validation	Usability or feasibility	Clinical outcome evaluation	Workflow implementation	Equity or safety assessment
Risk profile → follow-up prioritization	Moderate	Limited	Limited	Limited	Rare
Engagement phenotype → tailored coaching	Moderate	Moderate	Limited	Rare	Rare
Care needs profile → rehabilitation support	Limited	Limited	Limited	Rare	Rare
Digital twin → shared decision-making	Limited	Limited	Emerging	Rare	Rare
Large language model personalization → tailored education	Emerging	Emerging	Rare	Rare	Rare

^a^Evidence maturity ratings were assigned interpretively during synthesis and were not intended as formal risk-of-bias or certainty-of-evidence ratings. For each profile-to-action pathway, “moderate” indicated that several studies in the included evidence base reported relevant technical validation and at least 1 additional form of evaluation such as usability, feasibility, or clinical outcome assessment; “limited” indicated that evidence was present but sparse, indirect, or concentrated in early development or single-context studies; “emerging” indicated very recent or early evidence with few empirical evaluations; and “rare” indicated that few or no studies explicitly assessed that translational dimension. The ratings were derived from the study-level coding in Multimedia Appendix 3, including profiling concept, AI method, application focus, evaluation approach, nursing relevance level, and reported ethical or implementation issues.

### Validation, Evaluation, and Implementation Gaps

Evaluation approaches varied across studies. Algorithmic validation commonly used predictive metrics such as clustering fit, accuracy, or model discrimination [31,32]. However, technical performance alone did not demonstrate whether a profile was clinically useful or actionable for nurses. Some studies linked decision support or profiling outputs to care planning, identification of high-need patients, follow-up services, or postacute care needs [23,24,34].

A major gap concerned the validity and reproducibility of profiles. Some studies used data-driven validation, expert review, patient feedback, or human-centered design methods [29,30], but external validation, interrater consistency, and cross-system reproducibility were limited. Profile-to-strategy matching was also underdeveloped. Although some studies linked personas, contextual triggers, recommender systems, knowledge graphs, or causal models to tailored support [49,82], matching rules were rarely formalized or tested across patients, nurses, and settings.

Clinical and patient-reported outcome evaluations were less common than technical validation. Some studies suggested benefits for rehabilitation, decision quality, adherence, self-management, caregiver support, or patient experience [23,25,32]. However, the evidence was limited by few controlled studies, short follow-up, single-center designs, and heterogeneous outcomes. Usability, acceptability, patient experience, stakeholder involvement, and implementation issues, including workflow integration, staff training, accountability, interoperability, and sustainability, were inconsistently assessed.

Ethical and equity issues require greater attention. Profiling uses sensitive clinical, behavioral, and contextual data, raising concerns about privacy, consent, surveillance, stereotyping, and bias. Equity concerns are heightened when profiles incorporate language preference, health literacy, socioeconomic status, race, age, disability, or digital access. Multilingual AI care agents may reduce communication barriers [45], but studies of sociodemographic bias in LLM-generated nursing care highlight the need for fairness and safety evaluation [50]. LLMs also introduce risks of hallucination, outdated information, inconsistent advice, and unclear responsibility.

## Discussion

### Principal Findings

This scoping review mapped evidence on AI-enabled patient profiling for personalized nursing and health support. Patient profiling was used as an umbrella concept covering patient profiles, personas, digital phenotypes, segmentation, clinical subtypes, digital twins, and LLM-enabled personalization. Across these forms, an input-process-output logic was evident: patient-related data were processed by AI methods to generate representations that could support tailored education, self-management, rehabilitation, monitoring, decision support, and follow-up.

The most clinically relevant studies connected profiling outputs to specific nursing or health support actions, such as adherence messaging in cardiac rehabilitation [25], a rehabilitation nursing platform for spinal cord injury [46], digital twins for shared decision-making in knee osteoarthritis care [22], and personas for eHealth self-management design [30]. However, many studies remained at the stages of model development, segmentation, or technical validation. The central challenge is whether profiles can be interpreted, trusted, and acted on in nursing practice [83,84].

This review did not treat all AI-based prediction or digital health studies as patient profiling studies. Evidence was interpreted according to whether AI methods produced patient representations that could inform personalized nursing or health support. Technically accurate predictions are not equivalent to actionable profiles; profiling outputs must be interpretable, linked to care actions, and meaningful for nurses and patients [85,86].

### Comparison With Prior Work

Previous reviews of AI-based technologies in nursing have mapped broad categories of tools, including monitoring, prediction, decision support, and communication technologies, while noting that much of the evidence remains at an early stage of development [4]. Reviews of digital phenotyping and health care personas have examined mobile, wearable, and participatory representations separately [8,11]. This review differs by focusing on AI-enabled patient profiling as a mechanism that links heterogeneous patient data to personalized nursing or health support actions. By integrating adjacent concepts, this review shows that the key issue for nursing is not simply whether AI can generate classifications or predictions, but whether these representations can be interpreted, trusted, and translated into nursing actions. This distinction is important because many technically strong models do not yet provide clear guidance for education, monitoring, escalation, care planning, or evaluation of patient response.

### Implications for Clinical Nursing Practice

The findings are most useful for nursing as a data-to-action framework, rather than as evidence that AI-enabled profiling should already be adopted routinely. In the studies most directly relevant to nursing practice, profiles were used to interpret assessment data, identify high-need patients, tailor education or follow-up, and support rehabilitation, care planning, or decision support. In clinical nursing practice, profile outputs should therefore be linked to explicit actions: which assessment is triggered, which educational content is selected, how follow-up intensity is adjusted, when escalation is needed, and how patient response is evaluated.

In practice, AI-enabled patient profiles should be embedded in a nurse-mediated assessment-planning-implementation-evaluation-escalation pathway. During assessment, nurses should verify that profile inputs are complete, current, and consistent with bedside observations, patient-reported concerns, and contextual information. During planning, profile outputs should be translated into explicit nursing priorities, including symptom monitoring, education needs, self-management support, rehabilitation goals, discharge preparation, and follow-up intensity. During implementation, nurses may use profiles to tailor education, communication, coaching, reminders, care coordination, and referrals, while retaining responsibility for accepting, modifying, or overriding AI-generated recommendations. During evaluation, profile-informed actions should be assessed using nursing-sensitive and patient-centered outcomes, such as symptom burden, function, self-management capacity, patient understanding, adherence, and care continuity. Profiles indicating deterioration, high risk, inconsistent information, potential bias, or unclear accountability should trigger senior nursing review, multidisciplinary consultation, clinical escalation, or reassessment of the underlying data and algorithmic output.

Implementation also requires close attention to nursing workload and workflow. Profiling systems are more likely to be usable when they are embedded in electronic health records or existing digital documentation systems, provide concise explanations at the point of care, and avoid duplicative data entry, alert fatigue, or fragmented communication. Responsibility for accepting, modifying, or overriding AI-generated profile recommendations should be explicit and should include escalation procedures and documentation expectations. Patient-facing education generated from profiles should be explainable, readable, culturally appropriate, and consistent with local nursing guidance.

### Research and Implementation Gaps

Future research should strengthen conceptual clarity, profile validity, and profile-to-action pathways. Authors should specify the profile type, data sources, intended nursing or health support function, and the mechanism by which the profile informs care. Technical metrics alone are insufficient; validation should include expert assessment, patient feedback, external validation, and reproducibility testing when appropriate. Reporting should align with clinical AI guidance such as CONSORT (Consolidated Standards of Reporting Trials)-AI, SPIRIT (Standard Protocol Items: Recommendations for Interventional Trials)-AI, MI-CLAIM (Minimum Information about Clinical Artificial Intelligence Modeling), and DECIDE-AI (Developmental and Exploratory Clinical Investigations of Decision-support systems driven by Artificial Intelligence), which emphasize intended use, human-AI interaction, safety monitoring, and early-stage clinical evaluation [87-90].

Profile-to-strategy matching requires stronger nursing-specific methods. Many studies generated profiles without explaining how outputs translated into tailored nursing actions. Future work should make matching rules explicit through care pathways, rule tables, decision trees, recommender logic, or model explanations, and should test whether nurses can apply these rules consistently across shifts, systems, and sites. Intervention studies should evaluate nursing-sensitive outcomes, such as symptom burden, self-management capacity, function, quality of life, care continuity, readmission, patient engagement, and patient understanding, alongside implementation outcomes, including nurse workload, time burden, acceptability, usability, documentation burden, and sustainability.

Equity and safety evaluation should be operationalized in nursing contexts rather than addressed only as general AI ethics. Profiling systems should be assessed across language, health literacy, socioeconomic status, age, disability, culture, and digital access groups because these factors shape education, self-management, monitoring, and follow-up. LLM-enabled education or conversational support also requires safeguards for hallucination, inconsistent advice, and unclear responsibility. Cross-cultural applicability should be examined before profile categories, educational messages, or decision rules are transferred across health systems, languages, and nursing practice models.

### Strengths and Limitations

This review focused on patient profiling as a mechanism for personalized nursing and health support, rather than on a broad review of AI in nursing. It included diverse profiling concepts and used an input-process-output framework to organize evidence across data sources, methods, outputs, applications, and gaps. This approach clarified how patient data may be translated into tailored nursing or health support.

Several limitations should be noted. Although the search strategy included explicit patient profiling terms and adjacent patient representation concepts, terminology in this emerging field remains inconsistent. Therefore, some studies using uncommon or highly domain-specific labels for patient representation may still have been missed. Included studies varied in design, population, data sources, AI methods, and outcomes, which limited comparison. This scoping review did not assess intervention effectiveness, conduct a meta-analysis, or undertake formal risk-of-bias appraisal. Gray literature, preprints, conference abstracts, and protocol registers were not searched, and reference list chasing was not used as an additional source of study identification. Not using reference list chasing may have biased the review against older foundational informatics, recommender system, persona development, and clinical decision support studies that shaped later patient profiling frameworks but did not use the exact terminology captured in the database searches. Retrospective protocol registration may also limit perceived methodological transparency, although no changes were made to the final database sources, search date, core eligibility criteria, or main data-charting domains after registration. Finally, AI and LLM applications are developing rapidly, and new studies may appear after the final search date.

### Conclusions

Existing evidence describes AI-enabled patient profiling as a means of representing heterogeneous patient data for personalized nursing and health support. Profiling was operationalized through patient profiles, personas, digital phenotypes, patient segmentation, clinical subtypes, digital twins, and LLM-enabled personalization, with applications in education, self-management, rehabilitation, chronic disease management, remote monitoring, decision support, and follow-up.

However, prediction is not equivalent to actionable profiling. For nursing practice, profiles must be valid, interpretable, reproducible, equitable, and linked to clear care actions. Future research should prioritize profile validation, explicit profile-to-action pathways, nurse and patient involvement, equity assessment, and implementation evaluation in real-world nursing settings.

## Appendix

Multimedia Appendix 1 Full search strategies.

Multimedia Appendix 2 Data-charting form.

Multimedia Appendix 3 Study characteristics, analytic coding, and full-text exclusion reasons.

Multimedia Appendix 4 PRISMA-ScR checklist.

## Abbreviations

CBM: Chinese Biomedical Literature Database
CNKI: China National Knowledge Infrastructure
CONSORT: Consolidated Standards of Reporting Trials
DECIDE-AI: Developmental and Exploratory Clinical Investigations of Decision-support systems driven by Artificial Intelligence
LLM: large language model
MI-CLAIM: Minimum Information about Clinical Artificial Intelligence Modeling
PrEP: pre-exposure prophylaxis
PRISMA-ScR: Preferred Reporting Items for Systematic Reviews and Meta-Analyses Extension for Scoping Reviews
SPIRIT: Standard Protocol Items: Recommendations for Interventional Trials

## Data Availability

Data generated or analyzed during this review are available in Multimedia Appendices 1-4. These include the full search strategies, data-charting form, study characteristics, analytic coding, full-text exclusion reasons, and the completed PRISMA-ScR checklist.
