# Supplementary material for: AI-Enabled Patient Profiling for Personalized Nursing Care and Health Support: Scoping Review
Source: J Med Internet Res. 2026 Oct 29;28:e103362. doi: 10.2196/103362 (PMC13760325; doi:10.2196/103362)
Supplement: Supplementary file 2 [file jmir_v28i1e103362_app1.docx]

# Multimedia Appendix 1. Full search strategies.

Final search date: May 18, 2026.

Search strategies were developed using three concept blocks: artificial intelligence, patient profiling, and nursing or personalized care. Controlled vocabulary terms were used only where they directly corresponded to the original PubMed search concepts. Free-text terms were adapted across databases according to database-specific syntax and searchable fields. Chinese search terms were adapted to reflect commonly used Chinese terminology in nursing and digital health databases.

Search terms and keywords were organized into three concept blocks and are listed in full below for each database. No publication-date restriction was applied; searches covered each database from inception to May 18, 2026. Language limits were applied as appropriate to include Chinese- or English-language records. Publication-type restrictions were not applied during the database search where the database interface did not support an equivalent limit; reviews, protocols, editorials, commentaries, conference abstracts, preprints, and records with insufficient information for data charting were excluded during screening or full-text assessment according to the eligibility criteria. Quotation marks were used around exact phrases where supported by the database interface, and truncation was used for nurs* in English databases to capture nursing-related variants. Within each concept block, terms were combined with OR; the artificial intelligence, patient-profiling, and nursing/personalized-care blocks were then combined with AND. NOT was not used in the final search strings.

## Database yields recorded for the review

| Language | Database | Records identified, n |
| --- | --- | --- |
| Chinese | China National Knowledge Infrastructure (CNKI) | 10 |
| Chinese | Wanfang Data | 10 |
| Chinese | VIP Database | 5 |
| Chinese | Chinese Biomedical Literature Database | 4 |
| English | PubMed | 131 |
| English | CINAHL | 40 |
| English | Web of Science Core Collection | 707 |
| English | Embase | 265 |
| Total | All databases | 1172 |

## PubMed search strategy

| Search line | Search terms |
| --- | --- |
| 1 | "Generative Artificial Intelligence"[Mesh] OR "Large Language Models"[Mesh] OR "Artificial Intelligence"[Mesh] OR "Machine Learning"[Mesh] OR "Deep Learning"[Mesh] OR "Generative AI"[tiab] OR "large language model"[tiab] OR ChatGPT[tiab] OR "artificial intelligence"[tiab] OR "machine learning"[tiab] OR "deep learning"[tiab] |
| 2 | persona[tiab] OR "patient persona"[tiab] OR "user persona"[tiab] OR "patient profiling"[tiab] OR "patient segmentation"[tiab] OR "user modeling"[tiab] OR "patient typology"[tiab] |
| 3 | "Nursing Care"[Mesh] OR nurs*[tiab] OR "personalized care"[tiab] OR "individualized care"[tiab] OR "precision nursing"[tiab] OR "precision care"[tiab] OR "tailored intervention"[tiab] |
| 4 | 1 AND 2 AND 3 |
| 5 | Limit 4 to English language |

## CINAHL search strategy

| Search line | Search terms |
| --- | --- |
| S1 | (TI "Generative AI" OR AB "Generative AI" OR TI "large language model" OR AB "large language model" OR TI ChatGPT OR AB ChatGPT OR TI "artificial intelligence" OR AB "artificial intelligence" OR TI "machine learning" OR AB "machine learning" OR TI "deep learning" OR AB "deep learning") |
| S2 | (TI persona OR AB persona OR TI "patient persona" OR AB "patient persona" OR TI "user persona" OR AB "user persona" OR TI "patient profiling" OR AB "patient profiling" OR TI "patient segmentation" OR AB "patient segmentation" OR TI "user modeling" OR AB "user modeling" OR TI "patient typology" OR AB "patient typology") |
| S3 | (TI nurs* OR AB nurs* OR TI "personalized care" OR AB "personalized care" OR TI "individualized care" OR AB "individualized care" OR TI "precision nursing" OR AB "precision nursing" OR TI "precision care" OR AB "precision care" OR TI "tailored intervention" OR AB "tailored intervention") |
| S4 | S1 AND S2 AND S3 |
| S5 | Limit S4 to English language |

## Embase search strategy

| Search line | Search terms |
| --- | --- |
| 1 | 'Generative AI':ti,ab,kw OR 'large language model':ti,ab,kw OR ChatGPT:ti,ab,kw OR 'artificial intelligence':ti,ab,kw OR 'machine learning':ti,ab,kw OR 'deep learning':ti,ab,kw |
| 2 | persona:ti,ab,kw OR 'patient persona':ti,ab,kw OR 'user persona':ti,ab,kw OR 'patient profiling':ti,ab,kw OR 'patient segmentation':ti,ab,kw OR 'user modeling':ti,ab,kw OR 'patient typology':ti,ab,kw |
| 3 | nurs*:ti,ab,kw OR 'personalized care':ti,ab,kw OR 'individualized care':ti,ab,kw OR 'precision nursing':ti,ab,kw OR 'precision care':ti,ab,kw OR 'tailored intervention':ti,ab,kw |
| 4 | #1 AND #2 AND #3 |
| 5 | Limit #4 to English |

## Web of Science Core Collection search strategy

| Search line | Search terms |
| --- | --- |
| 1 | TS=("Generative AI" OR "large language model" OR ChatGPT OR "artificial intelligence" OR "machine learning" OR "deep learning") |
| 2 | TS=(persona OR "patient persona" OR "user persona" OR "patient profiling" OR "patient segmentation" OR "user modeling" OR "patient typology") |
| 3 | TS=(nurs* OR "personalized care" OR "individualized care" OR "precision nursing" OR "precision care" OR "tailored intervention") |
| 4 | #1 AND #2 AND #3 |
| 5 | Refined by: English language |

## Chinese database search strategy

| Search line | Search terms |
| --- | --- |
| 1 | （生成式人工智能 OR 生成式AI OR 大语言模型 OR ChatGPT OR 人工智能 OR 机器学习 OR 深度学习 OR 自然语言处理） |
| 2 | （画像 OR患者画像 OR 用户画像 OR 人物画像 OR 患者分型 OR 数字表型） |
| 3 | （护理 OR 个性化护理 OR 个体化护理 OR 精准护理 OR 差异化护理 OR 个性化照护 OR 精准照护 OR 分层管理 OR 精准干预 OR 患者护理计划 OR 护理决策支持） |
| 4 | #1 AND #2 AND #3 |
