# Supplementary material for: AI-Enabled Patient Profiling for Personalized Nursing Care and Health Support: Scoping Review
Source: J Med Internet Res. 2026 Oct 29;28:e103362. doi: 10.2196/103362 (PMC13760325; doi:10.2196/103362)
Supplement: Supplementary file 3 [file jmir_v28i1e103362_app2.docx]

# Multimedia Appendix 2. Data charting form.

Data were charted using a predefined form aligned with the review aim, the Population-Concept-Context framework, and the input-process-output analytical framework. The form was piloted on included studies and refined before formal data charting.

| Category | Data item | Definition/coding guidance |
| --- | --- | --- |
| Bibliographic information | Author(s); year; title; journal; country or region; DOI/URL | Basic citation information and study origin. Country/region refers to the study setting where reported. |
| Study characteristics | Study aim; design; setting; sample size or data size | Record whether the source was an empirical study, system development study, methodological study, feasibility/usability study, implementation study or mixed-methods study. |
| Population | Patient group; caregivers if applicable; disease/condition; age group | Include patients, health service users, caregivers or digital health users receiving health-related support. |
| Care context | Clinical nursing; rehabilitation; chronic disease management; patient education; self-management; digital health; decision support; follow-up care | Code all relevant contexts. Multiple codes were permitted when a study addressed more than one care context. |
| Profiling concept | Patient profile; patient persona; user persona; digital phenotype; patient segmentation; clinical subtype; digital twin; individualized patient model; personalized recommendation | Record the terminology used by the authors and map it to the closest profiling concept used in this review. |
| Data sources | Electronic health records; clinical indicators; patient-reported outcomes; questionnaires; mobile app data; web engagement data; wearable/sensor/IoT data; text/conversation data; multimodal data | Code all data sources used to construct, classify, validate or apply patient profiles. |
| AI/computational methods | Machine learning; clustering; latent profile analysis; deep learning; natural language processing; recommender system; knowledge graph; digital twin modeling; large language model; generative AI | Record the specific AI or computational method and broader method category. |
| Profiling output | Risk profile; care needs profile; behavioral phenotype; engagement phenotype; patient segment; clinical subtype; persona; digital twin; individualized recommendation | Record the form of patient representation generated or applied. |
| Application purpose | Personalized education; nursing assessment; intervention matching; self-management support; rehabilitation guidance; care planning; decision support; remote monitoring; follow-up care; shared decision-making | Record how the profiling output was used or intended to be used in nursing or health support. |
| Validation/evaluation | Algorithmic validation; profile validation; expert review; patient feedback; usability testing; feasibility evaluation; clinical outcomes; patient-reported outcomes; implementation outcomes | Record all evaluation approaches and outcomes. Note when only technical metrics were reported. |
| Nursing relevance | Assessment; education; monitoring; adherence support; rehabilitation nursing; continuity of care; patient engagement; shared decision-making | Describe the relevance of the study to clinical nursing or health support, even when the study was not nurse-led. |
| Ethical and implementation issues | Privacy; data security; bias; fairness; explainability; workflow integration; patient and nurse involvement; safety; accountability | Record reported concerns, limitations or implementation considerations. |
| Inclusion rationale | Reason for inclusion in the scoping review | Summarize how the study met the three inclusion components: AI/computational method, patient profiling-related concept, and nursing/health support context. |

Note. Multiple coding was permitted where one study involved more than one data source, AI method, profiling output, or application context.
