# Supplementary material for: AI-Enabled Patient Profiling for Personalized Nursing Care and Health Support: Scoping Review
Source: J Med Internet Res. 2026 Oct 29;28:e103362. doi: 10.2196/103362 (PMC13760325; doi:10.2196/103362)
Supplement: Supplementary file 4 [file jmir_v28i1e103362_app3.docx]

# Multimedia Appendix 3. Study characteristics, analytic coding, and full-text exclusion reasons.

Table S1 presents study-level characteristics, profiling concepts, AI methods, applications, evaluation approaches, and evidence relevance levels for the included studies.

## Table S1. Characteristics of included studies and coding for evidence synthesis

| Main-text reference No. | **Citation** | **Title** | **Journal (year)** | **Health support context** | **Profiling concept** | **AI/computational method** | **Profiling/application focus** | **Nursing relevance/evidence level** | **Identifier  (DOI/PMID/URL where available)** |
| --- | --- | --- | --- | --- | --- | --- | --- | --- | --- |
| 48 | Dai et al. (2024) | Construction and application of a spinal cord injury rehabilitation care platform based on patient portrait[In Chinese] | Chinese Journal of Nursing (2024) | Clinical nursing / care support | Patient profile / profiling | AI/computational personalization method | patient profile for rehabilitation | Level 1: direct nursing/health support evidence | doi.org/10.3761/j.issn.0254-1769.2024.22.001 |
| 29 | Tauro et al. (2025) | A novel data-driven approach for Personas validation in healthcare using self-supervised machine learning | Journal of Biomedical Informatics (2025) | Digital health / personalized health support | Patient/user persona; Patient segmentation / clinical subtype | machine learning; clustering/unsupervised learning | patient/user persona; patient segment/subgroup; personalized recommendation/support | Level 2: transferable health support evidence | doi:10.1016/j.jbi.2025.104815 |
| 28 | Rodriguez et al. (2024) | Leveraging Machine Learning to Develop Digital Engagement Phenotypes of Users in a Digital Diabetes Prevention Program: Evaluation Study | JMIR AI (2024) | Chronic disease / self-management | Patient segmentation / digital phenotype / subtype | machine learning; clustering/unsupervised learning | digital phenotype; patient segment/subgroup for adherence/engagement support | Level 3: methodological profiling evidence | doi:10.2196/47122 |
| 78 | Zheng et al (2025) | Exploration and practice of a whole-process intelligent health education model for day surgery [In Chinese] | Chinese Health Quality Management (2025) | Clinical nursing / care support | Patient profile / profiling | AI/computational personalization method | patient profile for education | Level 1: direct nursing/health support evidence | doi.org/10.13912/j.cnki.chqm.2025.32.4.05 |
| 37 | Abbasian et al. (2025) | Conversational health agents: a personalized large language model-powered agent framework | JAMIA Open (2025) | Digital health / personalized health support | LLM-enabled personalization | LLM/generative AI | personalized recommendation/support | Level 3: methodological profiling evidence | doi:10.1093/jamiaopen/ooaf067 |
| 40 | Adikari et al. (2024) | Is Mild Really Mild?: Generating Longitudinal Profiles of Stroke Survivor Impairment and Impact Using Unsupervised Machine Learning | Applied Sciences (2024) | Clinical nursing / care support | Patient profile / personalized support model | machine learning; clustering/unsupervised learning | patient profile for rehabilitation, monitoring | Level 1: direct nursing/health support evidence | doi:10.3390/app14156800 |
| 26 | Aharon et al. (2022) | Improving cardiac rehabilitation patient adherence via personalized interventions | PLOS ONE (2022) | Rehabilitation / functional recovery | Patient profile / user model | AI/computational personalization method | patient profile; personalized recommendation/support for rehabilitation, adherence/engagement support | Level 1: direct nursing/health support evidence | doi:10.1371/journal.pone.0273815 |
| 53 | Bai et al. (2025) | Detecting Sociodemographic Biases in the Content and Quality of Large Language Model–Generated Nursing Care: Cross-Sectional Simulation Study | Journal of Medical Internet Research (2025) | Clinical nursing / care support | Patient profile / personalized support model | LLM/generative AI | patient profile | Level 1: direct nursing/health support evidence | doi:10.2196/78132 |
| 25 | Bohanec et al. (2021) | HeartMan DSS: A decision support system for self-management of congestive heart failure | Expert Systems with Applications (2021) | Chronic disease / self-management | Patient profile / personalized support model | Decision support / recommender system | patient profile for self-management, decision support | Level 1: direct nursing/health support evidence | doi:10.1016/j.eswa.2021.115688 |
| 35 | Campagner et al. (2025) | Uncovering hidden subtypes in dementia: An unsupervised machine learning approach to dementia diagnosis and personalization of care | Journal of Biomedical Informatics (2025) | Older adult / long-term care | Patient segmentation / clinical subtype | machine learning; clustering/unsupervised learning | patient segment/subgroup | Level 2: transferable health support evidence | doi:10.1016/j.jbi.2025.104799 |
| 50 | Chen et al. (2025) | The digital intelligent precise nursing framework: theory development in health recommender system | BMC Nursing (2025) | Clinical nursing / care support | Personalized patient model / recommendation target | recommender system | personalized recommendation/support | Level 1: direct nursing/health support evidence | doi:10.1186/s12912-025-03830-2 |
| 33 | Chien et al. (2020) | A Machine Learning Approach to Understanding Patterns of Engagement With Internet-Delivered Mental Health Interventions | JAMA Network Open (2020) | Mental health / behavioral health | Patient profile / personalized support model | machine learning | patient profile for adherence/engagement support | Level 2: transferable health support evidence | doi:10.1001/jamanetworkopen.2020.10791 |
| 82 | Du et al. (2025) | Comparing Artificial Intelligence–Generated and Clinician-Created Personalized Self-Management Guidance for Patients With Knee Osteoarthritis: Blinded Observational Study | Journal of Medical Internet Research (2025) | Rehabilitation / functional recovery | Patient profile / user model | Artificial intelligence / computational modeling | patient profile; personalized recommendation/support for self-management | Level 1: direct nursing/health support evidence | doi:10.2196/67830 |
| 44 | Faro et al. (2020) | Comparison of a Collective Intelligence Tailored Messaging System on Smoking Cessation Between African American and White People Who Smoke: Quasi-Experimental Design | JMIR mHealth and uHealth (2020) | Clinical nursing / care support | Personalized patient model / recommendation target | Artificial intelligence / computational modeling | personalized recommendation/support for adherence/engagement support | Level 3: methodological profiling evidence | doi:10.2196/18064 |
| 88 | Fiorini et al. (2017) | Unsupervised Machine Learning for Developing Personalized Behavior Models Using Activity Data | Sensors (2017) | Older adult / long-term care | Patient segmentation / clinical subtype | machine learning; clustering/unsupervised learning | patient segment/subgroup; personalized recommendation/support for monitoring | Level 1: direct nursing/health support evidence | doi:10.3390/s17051034 |
| 86 | Goldstein et al. (2024) | Multi-Dimensional Validation of the Integration of Syntactic and Semantic Distance Measures for Clustering Fibromyalgia Patients in the Rheumatic Monitor Big Data Study | Bioengineering (2024) | Digital health / personalized health support | Patient segmentation / clinical subtype | NLP/text mining; machine learning; clustering/unsupervised learning | patient segment/subgroup; personalized recommendation/support | Level 2: transferable health support evidence | doi:10.3390/bioengineering11010097 |
| 46 | Guni et al. (2021) | Harnessing Machine Learning to Personalize Web-Based Health Care Content | Journal of Medical Internet Research (2021) | Older adult / long-term care | Patient profile / profiling | machine learning; recommender system | patient profile; personalized recommendation/support for education, self-management | Level 1: direct nursing/health support evidence | doi:10.2196/25497 |
| 22 | Hewner et al. (2023) | Identifying High-Need Primary Care Patients Using Nursing Knowledge and Machine Learning Methods | Applied Clinical Informatics (2023) | Clinical nursing / care support | Patient profile / personalized support model | machine learning | patient profile | Level 1: direct nursing/health support evidence | doi:10.1055/a-2048-7343 |
| 23 | Jayakumar et al. (2025) | Shared decision making using digital twins in knee osteoarthritis care: a randomized clinical trial of an AI-enabled decision aid versus education alone on decision quality, physical function, and user experience | eClinicalMedicine (2025) | Clinical nursing / care support | Digital twin / individualized model | digital twin modeling | digital twin/individual model for education, decision support | Level 1: direct nursing/health support evidence | doi:10.1016/j.eclinm.2025.103545 |
| 24 | Joymangul et al. (2023) | Homecare interventions as a Service model for Obstructive sleep Apnea: Delivering personalized phone call using patient profiling and adherence predictions | International Journal of Medical Informatics (2023) | Chronic disease / self-management | Patient profile / profiling | AI/computational personalization method | patient profile; personalized recommendation/support for monitoring, adherence/engagement support | Level 1: direct nursing/health support evidence | doi:10.1016/j.ijmedinf.2022.104935 |
| 85 | Kapse et al. (2025) | MediServe: An IoT-Enhanced Deep Learning Framework for Personalized Medication Management for Elderly Care | Computers, Materials & Continua (2025) | Older adult / long-term care | Patient profile / user model | machine learning | patient profile; personalized recommendation/support | Level 2: transferable health support evidence | doi:10.32604/cmc.2025.061981 |
| 34 | Kim et al. (2021) | Machine Learning Analysis to Identify Digital Behavioral Phenotypes for Engagement and Health Outcome Efficacy of an mHealth Intervention for Obesity: Randomized Controlled Trial | Journal of Medical Internet Research (2021) | Chronic disease / self-management | Patient segmentation / digital phenotype / subtype | machine learning; clustering/unsupervised learning | digital phenotype; patient segment/subgroup for adherence/engagement support | Level 2: transferable health support evidence | doi:10.2196/27218 |
| 73 | Kowlagi et al. (2025) | An Unsupervised Learning Approach for Multimodal Low Back Pain Stratification | Spine (2025) | Rehabilitation / functional recovery | Patient profile / personalized support model | machine learning; clustering/unsupervised learning | patient profile | Level 3: methodological profiling evidence | doi:10.1097/BRS.0000000000005593 |
| 45 | Li et al. (2020) | A Personalized Voice-Based Diet Assistant for Caregivers of Alzheimer Disease and Related Dementias: System Development and Validation | Journal of Medical Internet Research (2020) | Chronic disease / self-management | Patient profile / user model | AI/computational personalization method | patient profile; personalized recommendation/support | Level 2: transferable health support evidence | doi:10.2196/19897 |
| 30 | Li et al. (2025) | Discovering patient groups in sequential electronic healthcare data using unsupervised representation learning | BMC Medical Informatics and Decision Making (2025) | Digital health / personalized health support | Patient profile / personalized support model | machine learning; clustering/unsupervised learning | patient profile | Level 2: transferable health support evidence | doi:10.1186/s12911-024-02812-9 |
| 87 | Liao et al. (2025) | Enhancing Patient Outcomes in Head and Neck Cancer Radiotherapy: Integration of Electronic Patient-Reported Outcomes and Artificial Intelligence-Driven Oncology Care Using Large Language Models | Cancers (2025) | Cancer care / symptom management | Patient segmentation / clinical subtype; LLM-enabled personalization | LLM/generative AI | patient segment/subgroup; personalized recommendation/support for education, monitoring, adherence/engagement support | Level 1: direct nursing/health support evidence | doi:10.3390/cancers17142345 |
| 62 | Linardon et al. (2025) | Integrating Artificial Intelligence and Smartphone Technology to Enhance Personalized Assessment and Treatment for Eating Disorders | International Journal of Eating Disorders (2025) | Mental health / behavioral health | Patient profile / user model | Artificial intelligence / computational modeling | patient profile; personalized recommendation/support | Level 3: methodological profiling evidence | doi:10.1002/eat.24468 |
| 42 | Lima et al. (2023) | Discovering Behavioral Patterns Using Conversational Technology for In-Home Health and Well-Being Monitoring | IEEE Internet of Things Journal (2023) | Clinical nursing / care support | Patient profile / personalized support model | AI/computational personalization method | patient profile for monitoring | Level 1: direct nursing/health support evidence | doi:10.1109/JIOT.2023.3290833 |
| 79 | Lopez-Castroman et al. (2021) | Psychiatric Profiles of eHealth Users Evaluated Using Data Mining Techniques: Cohort Study | JMIR Mental Health (2021) | Mental health / behavioral health | Patient profile / personalized support model | AI/computational personalization method | patient profile | Level 3: methodological profiling evidence | doi:10.2196/17116 |
| 70 | Marfoglia et al. (2026) | A knowledge graph-driven framework for deploying AI-powered patient digital twins | Future Generation Computer Systems (2026) | Digital health / personalized health support | Digital twin / individualized model | knowledge graph; digital twin modeling | digital twin/individual model | Level 3: methodological profiling evidence | doi:10.1016/j.future.2026.108380 |
| 67 | Midelet et al. (2021) | Hidden Markov model segmentation to demarcate trajectories of residual apnea-hypopnoea index in CPAP-treated sleep apnea patients to personalize follow-up and prevent treatment failure | EPMA Journal (2021) | Clinical nursing / care support | Patient segmentation / clinical subtype | clustering/unsupervised learning | patient segment/subgroup for follow-up | Level 1: direct nursing/health support evidence | doi:10.1007/s13167-021-00264-z |
| 75 | Momand et al. (2025) | Building Digital Twins for Elderly Care: An End-to-End Framework From Data Acquisition to Modeling | IEEE Access (2025) | Older adult / long-term care | Digital twin / individualized model | digital twin modeling | digital twin/individual model | Level 2: transferable health support evidence | doi:10.1109/ACCESS.2025.3607603 |
| 68 | Naseri et al. (2022) | Adaptive and personalized user behavior modeling in complex event processing platforms for remote health monitoring systems | Artificial Intelligence in Medicine (2022) | Remote monitoring / home care | Patient profile / user model | AI/computational personalization method | patient profile; personalized recommendation/support for monitoring | Level 1: direct nursing/health support evidence | doi:10.1016/j.artmed.2022.102421 |
| 84 | Sadasivam et al. (2016) | Impact of a Collective Intelligence Tailored Messaging System on Smoking Cessation: The Perspect Randomized Experiment | Journal of Medical Internet Research (2016) | Clinical nursing / care support | Personalized patient model / recommendation target | Artificial intelligence / computational modeling | personalized recommendation/support for adherence/engagement support | Level 3: methodological profiling evidence | doi:10.2196/jmir.6465 |
| 72 | Sang et al. (2025) | A smart community interactive art therapy platform based on multimodal computer graphics and resilient artificial intelligence for home-based elderly care | Scientific Reports (2025) | Older adult / long-term care | Patient profile / personalized support model | Artificial intelligence / computational modeling | patient profile for monitoring | Level 2: transferable health support evidence | doi:10.1038/s41598-025-27129-z |
| 77 | Schillinger et al. (2021) | Employing computational linguistics techniques to identify limited patient health literacy: Findings from the ECLIPPSE study | Health Services Research (2021) | Digital health / personalized health support | Patient profile / personalized support model | Natural language processing / text mining | patient profile | Level 3: methodological profiling evidence | doi:10.1111/1475-6773.13560 |
| 66 | Spinsante et al. (2023) | Clinically-validated technologies for assisted living: The vINCI project | Journal of Ambient Intelligence and Humanized Computing (2023) | Digital health / personalized health support | Patient profile / personalized support model | AI/computational personalization method | patient profile | Level 3: methodological profiling evidence | doi:10.1007/s12652-021-03419-y |
| 76 | Tang et al. (2026) | An AI-Driven Multimodal Smart Home Platform for Continuous Monitoring and Assistance in Post-Stroke Motor Impairment | IEEE Transactions on Neural Systems and Rehabilitation Engineering (2026) | Rehabilitation / functional recovery | Patient profile / personalized support model | Sensor/IoT analytics + machine learning | patient profile for monitoring | Level 1: direct nursing/health support evidence | doi:10.1109/TNSRE.2025.3645093 |
| 90 | Tang et al. (2021) | A Two-Echelon Responsive Health Analytic Model for Triggering Care Plan Revision in Geriatric Care Management: | Journal of Organizational and End User Computing (2021) | Clinical nursing / care support | Patient profile / personalized support model | Artificial intelligence / computational modeling | patient profile for monitoring | Level 1: direct nursing/health support evidence | doi:10.4018/JOEUC.289224 |
| 83 | Varidel et al. (2025) | Causal AI Recommendation System for Digital Mental Health: Bayesian Decision-Theoretic Analysis | Journal of Medical Internet Research (2025) | Mental health / behavioral health | Personalized patient model / recommendation target | recommender system | personalized recommendation/support for decision support | Level 1: direct nursing/health support evidence | doi:10.2196/71305 |
| 80 | Wang et al. (2024) | Promoting Personalized Reminiscence Among Cognitively Intact Older Adults Through an AI-Driven Interactive Multimodal Photo Album: Development and Usability Study | JMIR Aging (2024) | Older adult / long-term care | Patient profile / user model | Artificial intelligence / computational modeling | patient profile; personalized recommendation/support | Level 3: methodological profiling evidence | doi:10.2196/49415 |
| 89 | Xiao et al. (2022) | Recommendation of Healthcare Services Based on an Embedded User Profile Model: | International Journal on Semantic Web and Information Systems (2022) | Clinical nursing / care support | Patient profile / profiling | machine learning; recommender system | patient profile; personalized recommendation/support | Level 2: transferable health support evidence | doi:10.4018/IJSWIS.313198 |
| 39 | Yang et al. (2025) | Harnessing an Artificial Intelligence–Based Large Language Model With Personal Health Record Capability for Personalized Information Support in Postsurgery Myocardial Infarction: Descriptive Qualitative Study | Journal of Medical Internet Research (2025) | Digital health / personalized health support | LLM-enabled personalization | LLM/generative AI | personalized recommendation/support | Level 1: direct nursing/health support evidence | doi:10.2196/68762 |
| 52 | Yang et al. (2025) | Personalized Causal Graph Reasoning for LLMs: An Implementation for Dietary Recommendations | IEEE Journal of Biomedical and Health Informatics (2025) | Chronic disease / self-management | LLM-enabled personalization | LLM/generative AI; recommender system | personalized recommendation/support | Level 3: methodological profiling evidence | doi:10.1109/JBHI.2025.3624144 |
| 32 | Zhao et al. (2025) | User Personas for eHealth Regarding the Self-Management of Depressive Symptoms in People Living With HIV: Mixed Methods Study | Journal of Medical Internet Research (2025) | Mental health / behavioral health | Patient/user persona | AI/computational personalization method | patient/user persona for self-management | Level 1: direct nursing/health support evidence | doi:10.2196/56289 |
| 20 | An et al. (2021) | Machine learning‐based patient classification system for adult patients in intensive care units: A cross‐sectional study | Journal of Nursing Management (2021) | Digital health / personalized health support | Patient profile / personalized support model | machine learning | patient profile | Level 2: transferable health support evidence | doi:10.1111/jonm.13284 |
| 64 | Fagherazzi et al. (2024) | Heterogeneity of glycaemic phenotypes in type 1 diabetes | Diabetologia (2024) | Chronic disease / self-management | Patient segmentation / digital phenotype / subtype | clustering/unsupervised learning | digital phenotype; patient segment/subgroup | Level 3: methodological profiling evidence | doi:10.1007/s00125-024-06179-4 |
| 74 | Hors-Fraile et al. (2019) | Opening the Black Box: Explaining the Process of Basing a Health Recommender System on the I-Change Behavioral Change Model | IEEE Access (2019) | Digital health / personalized health support | Personalized patient model / recommendation target | recommender system | personalized recommendation/support | Level 3: methodological profiling evidence | doi:10.1109/ACCESS.2019.2957696 |
| 43 | Huang et al. (2025) | Developing nurse‐accessible hypertension prediction tools for low‐income populations: A comparative analysis of machine learning algorithms with SHAP interpretation | International Journal of Nursing Practice (2025) | Clinical nursing / care support | Patient profile / personalized support model | machine learning | patient profile for decision support | Level 1: direct nursing/health support evidence | doi:10.1111/ijn.70060 |
| 38 | Jelic et al. (2025) | Evaluating the clinical effectiveness and patient experience of a large language model–based digital tool for home-based blood pressure management: Mixed methods study | JMIR mHealth and uHealth (2025) | Clinical nursing / care support | Patient profile / personalized support model | LLM/generative AI | patient profile for monitoring | Level 1: direct nursing/health support evidence | doi:10.2196/68361 |
| 49 | Kumar et al. (2025) | Feature selection for physical activity prediction using ecological momentary assessments to personalize intervention timing: Longitudinal observational study | JMIR mHealth and uHealth (2025) | Older adult / long-term care | Patient profile / user model | Sensor/IoT analytics + machine learning | patient profile; personalized recommendation/support for monitoring | Level 1: direct nursing/health support evidence | doi:10.2196/57255 |
| 31 | Lee et al. (2025) | Development of personas and journey maps for artificial intelligence agents supporting the use of health big data: Human-centered design approach | JMIR Formative Research (2025) | Digital health / personalized health support | Patient/user persona | Artificial intelligence / computational modeling | patient/user persona | Level 1: direct nursing/health support evidence | doi:10.2196/67272 |
| 63 | Liao et al. (2025) | Enhancing PrEP adherence through person‐centered mobile app interventions: A real‐world data and machine learning approach using UPrEPU among gay, bisexual and other men who have sex with men in taiwan | Journal of the International AIDS Society (2025) | Chronic disease / self-management | Patient profile / personalized support model | machine learning | patient profile for self-management, adherence/engagement support | Level 1: direct nursing/health support evidence | doi:10.1002/jia2.70033 |
| 71 | Pokhrel Bhattarai et al. (2025) | Comprehensive analysis of heart failure subtypes presenting at emergency department for acute heart failure management | Journal of Emergency Nursing (2025) | Chronic disease / self-management | Patient segmentation / clinical subtype | clustering/unsupervised learning | patient segment/subgroup | Level 3: methodological profiling evidence | doi:10.1016/j.jen.2025.03.002 |
| 21 | Chae et al. (2025) | Developing a clinical decision support framework for integrating predictive models into routine nursing practices in home health care for patients with heart failure | Journal of Nursing Scholarship (2025) | Clinical nursing / care support | Patient profile / user model | Decision support / recommender system | patient profile; personalized recommendation/support for monitoring, decision support | Level 1: direct nursing/health support evidence | doi:10.1111/jnu.13030 |
| 69 | Chew et al. (2025) | Momentary dietary lapse prediction for obesity management: Developing the eating behavior lapse inventory survey singapore (eBLISS) and a machine learning lapse prediction model | Computer Methods and Programs in Biomedicine (2025) | Chronic disease / self-management | Patient profile / personalized support model | machine learning | patient profile for adherence/engagement support | Level 1: direct nursing/health support evidence | doi:10.1016/j.cmpb.2025.108880 |
| 41 | Fritz et al. (2025) | Detecting older adults’ behavior changes during adverse external events using ambient sensing: Longitudinal observational study | JMIR Nursing (2025) | Older adult / long-term care | Patient profile / personalized support model | Sensor/IoT analytics + machine learning | patient profile for monitoring | Level 1: direct nursing/health support evidence | doi:10.2196/69052 |
| 81 | Hilberger et al. (2025) | Design of a mobile app and a clinical trial management system for cognitive health and dementia risk reduction: User-centered design approach | JMIR Aging (2025) | Older adult / long-term care | Patient profile / user model | Artificial intelligence / computational modeling | patient profile; personalized recommendation/support | Level 2: transferable health support evidence | doi:10.2196/66660 |
| 36 | Leung et al. (2025) | Profiling unmet post–acute care needs of an inpatient population in hong kong: Can real-world data and machine learning algorithms bring precision to tertiary prevention in the community? | Hong Kong Medical Journal (2025) | Digital health / personalized health support | Patient profile / user model | machine learning | patient profile | Level 2: transferable health support evidence | doi:10.12809/hkmj2411474 |
| 65 | Li et al. (2026) | Novel bayesian nonparametric unsupervised learning approach to precision symptom management in cancer survivors: A re-analysis of a comparative effectiveness trial | Journal of Behavioral Medicine (2026) | Clinical nursing / care support | Patient segmentation / clinical subtype | machine learning; clustering/unsupervised learning | patient segment/subgroup; personalized recommendation/support | Level 1: direct nursing/health support evidence | doi:10.1007/s10865-025-00621-7 |
| 47 | Bhimani et al. (2025) | Using a multilingual AI care agent to reduce disparities in colorectal cancer screening for higher fecal immunochemical test adoption among spanish-speaking patients: Retrospective analysis | Journal of Medical Internet Research (2025) | Cancer care / symptom management | Patient profile / personalized support model | Artificial intelligence / computational modeling | patient profile | Level 2: transferable health support evidence | doi:10.2196/71211 |

Note. The first column now reports the corresponding main-text reference number for each included study, so that Table S1 can be read together with the revised manuscript reference list. Evidence relevance level was assigned according to the degree of direct relevance to nursing or health support. Level 1 indicates direct nursing or patient-care support evidence; Level 2 indicates transferable health support evidence; Level 3 indicates methodological profiling evidence with plausible relevance to personalized nursing or health support.

## Full-text exclusion reasons

| Reason for exclusion | Number of reports, n |
| --- | --- |
| No profiling concept | 12 |
| Prediction only | 5 |
| No AI method | 5 |
| Not nursing support | 11 |
| Ineligible publication type | 7 |
| Insufficient information | 5 |
| Total | 45 |
